# Health outcomes in chronic kidney disease patients with cognitive impairment or dementia: a global collaborative analysis

**DOI:** 10.1093/ckj/sfae401

**Published:** 2024-12-11

**Authors:** Lino Merlino, Francesco Rainone, Rajkumar Chinnadurai, Gema Hernandez, James Tollitt, Graziana G Battini, Paolo M Colombo, Marco Trivelli, Stuart Stewart, Ross A Dunne, Philip A Kalra

**Affiliations:** Faculty of Biology, Medicine and Health, School of Medical Sciences, University of Manchester, Oxford Road, Manchester, UK; Vimercate Hospital, ASST Brianza, Vimercate, Italy; Donal O'Donoghue Renal Research Centre, Northern Care Alliance NHS Foundation Trust, Salford Royal Hospital, Stott Lane, Salford, UK; Donal O'Donoghue Renal Research Centre, Northern Care Alliance NHS Foundation Trust, Salford Royal Hospital, Stott Lane, Salford, UK; Donal O'Donoghue Renal Research Centre, Northern Care Alliance NHS Foundation Trust, Salford Royal Hospital, Stott Lane, Salford, UK; TriNetX Europe NV, Kortrijksesteenweg 214 b3, 9830 Sint-Martens-Latem, Belgium; Donal O'Donoghue Renal Research Centre, Northern Care Alliance NHS Foundation Trust, Salford Royal Hospital, Stott Lane, Salford, UK; Vimercate Hospital, ASST Brianza, Vimercate, Italy; Vimercate Hospital, ASST Brianza, Vimercate, Italy; Vimercate Hospital, ASST Brianza, Vimercate, Italy; Donal O'Donoghue Renal Research Centre, Northern Care Alliance NHS Foundation Trust, Salford Royal Hospital, Stott Lane, Salford, UK; Rochdale Care Organisation, Northern Care Alliance NHS Foundation Trust, Greater Manchester, UK; Centre for Primary Care & Health Services Research, University of Manchester, Manchester, UK; Greater Manchester Dementia Research Centre, Greater Manchester Mental Health Foundation Trust, Manchester, UK; Geoffrey Jefferson Brain Research Centre, University of Manchester, UK; Faculty of Biology, Medicine and Health, School of Medical Sciences, University of Manchester, Oxford Road, Manchester, UK

**Keywords:** chronic kidney disease, cognitive impairment, dementia, health outcome, TriNetX

## Abstract

**Background and hypothesis:**

Mild cognitive impairment and dementia (CI) are common in patients with CKD. We aim to clarify whether and how CKD and CI coexistence increases adverse health outcomes.

**Methods:**

This retrospective observational cohort study was conducted on CKD patients (stages 3–5) from the TriNetX platform. CKD patients with and without pre-existing CI were included from 115 healthcare organizations, and their outcomes were compared. The two cohorts were propensity score matched (PSM) for age, sex, ethnicity, comorbidities, BMI, blood parameters, and medications. The proportional hazard assumption was tested with a 95% confidence interval. Kaplan–Meier analysis was used to calculate survival probability. Outcomes were included from 1 day after the CKD diagnosis until 10 years afterwards.

**Results:**

We identified 533 772 CKD patients, and 8184 had co-existent CI. Two cohorts of 8170 PSM patients each were generated. The mean age was 60.5 ± 7.0 years and the eGFR was 52.1±19 mL/min. Mean follow-up was 23.2 months. CKD patients with CI had higher all-cause mortality (18.5% vs 12.6%), higher risk of cerebrovascular disease (11.3% vs 6.9%), transient cerebral ischemic attacks (2.7% vs 1.6%), hypotension (16.5%–12.5%), malnutrition (6.7% vs 4.0%), pneumonia (10.7% vs 7.9%), urinary infections (13.2% vs 9.3%), encephalopathy (9.9% vs 5.0%), mood disorders (13.6% vs 9.7%), psychosis (9.8% vs 4.6%), and epilepsy (4.3% vs 1.5%). Higher use of antidepressants (26.3% vs 16.3%), anticonvulsants (19.5% vs 15.1%), antipsychotics (18.6% vs 9.1%), anticholinesterase (5.6% vs 0.1%), and benzodiazepines (30.6% vs 26.6%) was noted in those with CI. All these findings were statistically significant.

**Conclusion:**

Despite the limitations of a retrospective study, real-world data demonstrate that concomitant CI is a decisive risk factor for higher mortality and increased adverse outcomes in patients with CKD. These results highlight the need for routine comprehensive cognitive assessments in patients at any stage of CKD.

## Graphical Abstract

**Graphical Abstract:**
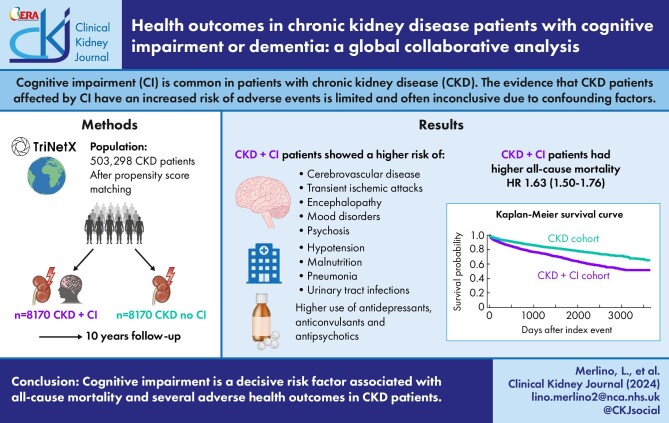

KEY LEARNING POINTS **What was known:**

- Simultaneous comorbidity of cognitive impairment (CI) and CKD reduces life expectancy. However, previous studies focused on patients with late CKD and/or dialysis, and had heterogeneous dementia onset.

**This study adds:**

- Specific adverse outcomes were identified during a long follow-up period of 10 years in a vast cohort of patients. Dementia onset of <5 years from CKD diagnosis avoids confounding factors due to a long course of the disease. A population younger than 70 years on enrolment avoids frailty due to ageing.

**Potential impact:**

- CI is a decisive risk factor in patients with CKD, even in its early stages. Neuropsychological evaluation must become a routine tool for CKD patients to identify CI. Therapy optimization, prevention of infections, and nutritional reviews to avoid sarcopenia are necessary to ameliorate outcomes in CKD + CI.

## INTRODUCTION

CKD is defined by an eGFR of <60 mL/min/1.73 m^2^ on at least two occasions 90 days apart [1]. Due to its chronic nature, this condition develops slowly and often progresses in stages, ranging from mild to severe CKD [1], with an estimated global prevalence between 8% and 16% [2]. CKD is associated with an increased risk of cardiovascular disease, all-cause mortality, and decreased quality of life [3].

Dementia is defined as progressive multidomain cognitive impairment (CI) causing a decline in instrumental activities of daily living. The most common causes are vascular dementia and Alzheimer's disease [4]. The prevalence of dementia is estimated to be ∼6%–8% after 65 years of age and rises to 20%–30% in subjects older than 85 years in the general population of Europe [5]. By 2050, ∼22% of the world's population aged >60 years will be affected [4].

Mild CI is an intermediate state between normal cognition and dementia that is challenging to diagnose [6]. We grouped vascular dementia, Alzheimer's disease, and mild CI together under the rubric ‘cognitive impairment’.

CKD and CI share a common genetic background [6] and risk factors, such as ageing, diabetes, cardiovascular disease, and hypertension, which frequently coexist, amplify, and confound each other [7]. CKD is associated with an increased risk of CI [8]. Reduction in the eGFR is directly related to a reduction in global cognitive performance [9]. Several studies proposed a putative direct effect of CKD as an independent risk factor for CI [10].

This was an aetiological study aiming to verify if patients with CKD stages 3–5 with a diagnosis of CI have different risk of death, cardiovascular outcomes, infections, medication burden, and prevalence of mental health diagnoses in comparison with patients with CKD stages 3–5 without a CI diagnosis.

## MATERIALS AND METHODS

### Study design and setting

This was a retrospective, observational cohort study of CKD patients (stages 3−5) as per the kidney disease improving global outcomes classification [11]. The data source used was the TriNetX platform [12]. Data for this study were accessed via the TriNetX Global Collaborative Network (GCN) [13] and analysed using the TriNetX built-in query builder.

TriNetX is a GCN that integrates anonymized electronic medical records (EMRs) of patients from >150 primary and secondary healthcare institutions worldwide, including academic medical centres, specialty hospitals, physician practices, and community hospitals, collectively called healthcare organizations (HCOs), providing data from uninsured and insured patients.

Available data span multiple geographic regions, age groups, and income levels. TriNetX continuously aggregates information directly from EMR systems. Data for analysis include patient demographics, diagnosis history, medications, procedures, and laboratory results [14]. All data collection, processing, and transmission were done in compliance with all data protection laws applicable to the contributing HCOs, including the EU Data Protection Law Regulation 2016/679, the General Data Protection Regulation on the protection of natural persons about the processing of personal data, and the US Health Insurance Portability and Accountability Act. The TriNetX is a distributed network, and analytics are performed at HCOs with only aggregate results being returned to the platform. All diagnoses were identified using the International Classification of Diseases, Tenth Revision and clinical modification codes (ICD10-CM), procedures were identified using The International Classification of Diseases, Tenth Revision, Procedure Coding System, ICD-10-PCS, or Current Procedural Terminology (CPT). Medications were identified using the Veterans Affairs National Formulary (NLM-VA). Laboratory test results were identified using the Logical Observation Identifiers Names and Codes (LOINC). Due to the anonymous nature of the data, informed consent was waived.

### Patient population

We conducted our study on two cohorts of patients aged between 18 and 70 years, all with CKD stages 3–5, who had never been on dialysis or had any kind of transplant. Patients with an eGFR <5 mL/min on the latest blood tests were excluded.

Cohort 1 (CKD + CI) included patients who also had a diagnosis of mild CI or dementia made a maximum of 5 years before the diagnosis of CKD (mild CI, vascular dementia, Alzheimer's disease). We grouped ICD 10CM codes F01, F02, and F03 for dementia, Alzheimer's disease (G30), and mild CI (G31.84) together under the rubric ‘cognitive impairment’.

Cohort 2 (CKD no CI) included patients never diagnosed with CI (mild CI, Alzheimer's disease, dementia in other diseases or unspecified dementia, alcohol dependence with alcohol-induced persisting dementia, frontotemporal dementia, or vascular dementia). See the cohorts’ definitions in the supplementary materials.

The index date for each patient within a cohort was the day the patient was first diagnosed with CKD stage 3, 4, or 5, which defined when each patient entered the analysis. The report was generated by the TriNetX platform on 7 March 2024. We included events that occurred up to 20 years ago. Patients whose diagnosis occurred ≥20 years ago were excluded.

### Pre-specified outcome

In addition to the classic causes of mortality and morbidity, we researched those who are typical of patients suffering from CI [15–17].

Outcome measures were captured within a 10-year window after diagnosis of CKD. Primary outcome measures were (in brackets) the ICD-10CM or the NLM-VA:

1. Deceased or ill-defined and unknown cause of mortality (R99).
2. Ischaemic heart diseases (I20-I25).
3. Arrhythmia: atrial fibrillation and flutter (I48), tachycardia (R00.0), ventricular tachycardia (I47.2), supraventricular tachycardia (I 47.1), or bradycardia (R00.1).
4. Pulmonary embolism (I26).
5. Pneumonia (J18) or pneumonia due to inhalation (J69.0).
6. Stroke (I60–I69).
7. Transitory cerebral ischaemic attacks (G45).
8. Hypotension (I95) or orthostatic hypotension (I95.1)
9. Malnutrition(E40–E46) or sarcopenia (M62.84).
10. Type 2 diabetes (E11).
11. Acute kidney failure (N17).
12. Cystitis (N30), urinary tract infection (N39.0), or acute pyelonephritis (N10).
13. Mood disorders (F30–F39).
14. Anxiety, dissociative, and other non-psychotic mental disorders (F40–F48).
15. Alcohol abuse (F10.1).
16. Encephalopathy: metabolic encephalopathy (G93.41), unspecified encephalopathy (G93.40), other disorders resulting from impaired renal tubular function (N25.89), or toxic encephalopathy (G92).
17. Epilepsy (G40).
18. Sleep disorders (G47).
19. Psychosis: somnolence (R40.0), disorientation (R41.0), psychosis not due to a substance (F29), major depressive disorder, severe with psychotic symptoms (F33.3), or major depressive disorder, single episode, severe with psychotic features (F32.3).
20. Antidepressants (CN600).
21. Anticonvulsants (CN400).
22. Antipsychotics (CN700).
23. Anticholinesterase: donepezil (NLM RXNORM 135447), rivastigmine (NLM RXNORM: 183379), or galantamine (NLM RXNORM 4637).
24. Benzodiazepine or derivates (CN30) or (NLM ATC: N05CD) or (NLM ATC: N05BA).
25. Angiotensin II receptor blockers (NLM ATC: C09C) or angiotensin-converting enzyme inhibitors (NLM ATC: C09A).

### Covariates and propensity score matching

To reduce the effect of confounders, we applied propensity score matching (PSM). We used the TriNetX built-in algorithm for PSM based on 1:1 nearest-neighbour matching with a margin of 0.1 SD. For matching, we included the age at index event, ethnicity, gender, comorbidities, nicotine dependence, BMI, eGFR [calculated by creatinine-based formula (MDRD); mL/min/1.73 m^2^], albumin, cholesterol, and use of CNS or cardiovascular medication and socioeconomic status. After PSM, 8170 patients were selected from each cohort for the study.

### Baseline characteristics of the study subjects

PSM was performed on 32 characteristics (ICD-10 codes used are reported in brackets).

For the demographic and lifestyle characteristics, patients were matched on age at the diagnosis of CKD, sex, race (White, Black or African American, Asian) and ethnicity (Hispanic-Latino, not Hispanic-Latino, Unknown ethnicity), nicotine dependence (F17), BMI, factors influencing health status and frequent contact with health services (Z00–Z99) comprehending health hazards due to socioeconomic circumstances, external causes of morbidity (V00–Y99), injury and poisoning (S00–T88).

Patients were also matched for endocrine and nutritional-metabolic diseases (E00–E89), diseases of the circulatory system (I00–I99), digestive disorders (K00–K95), respiratory disorders (J00–J99), genitourinary disorders (N00–N99), and nervous disorders (G00–G99), such as sleep disorders, epilepsy, headache, encephalopathy, polyneuropathies, and extrapyramidal movement disorders. We also matched for diseases of the blood and immune systems (D50–D89), diseases of the musculoskeletal-connective tissue system (M00–M99), neoplasms (C00–D49), infectious diseases (A00–B99), and mental disorders (F01–F99).

Patients were matched for medication use of CNS (CN000) and cardiovascular medications (CV000) like antilipaemic agents, β-blockers, antiarrhythmics, diuretics, calcium channel blockers, and ACE/RAS inhibitors.

To increase the homogeneity of the cohorts, they were also matched for levels of serum albumin (g/dL), cholesterol (mg/dL), and haemoglobin (g/dL), and eGFR.

### Statistical analysis

All data processing was conducted using the TriNetX built-in algorithms. Numerical baseline characteristics are presented as mean and standard deviation. Categorical characteristics are presented as the number of patients and percentage of the cohort. The *P*-value was calculated with a *t*-test for continuous variables and Fisher's exact test for categorical variables. We compared the outcomes 10 years after the index event (diagnosis of CKD) with measures of association and survival with the Compare Outcomes Analytic and Measures of Association and Survival tools integrated into the platform. Risks and hazard ratios with 95% CI are presented. We excluded patients from the analysis who had outcomes before the time window.

Kaplan–Meier analysis was used to estimate the probability of the outcome at daily temporal resolution. Patients were removed from the analysis (censored) after the last entry in their records. The log-rank test, hazard ratio calculation, and the test for proportionality assumptions were performed [18].

## RESULTS

For cohort 1 (CKD + CI), 115 HCOs were queried, and 115 HCOs responded. A total of 80 providers responded with patients. The final cohort included 8184 patients: 32% with mild CI, 12% with vascular dementia, 32% with unspecified dementia, 15% with dementia in other diseases, 8% with Alzheimer's disease, and 1% with frontotemporal dementia.

For cohort 2 (CKD no CI) 115 HCOs were queried and 115 HCOs responded. A total of 98 providers responded with patients. This resulted in the availability of 523 772 CKD patients with no CI, who were then available for PSM with cohort 1. The number of individuals at each stage of the study design is reported in Table 1.

**Table 1:** Individuals and healthcare organizations at each stage of the study design.

	Patients		Healthcare organizations	
Network	142 534 773		115	
CKD stage 3–5 eGFR >5 mL/min	1 049 770		98	
Age 18–70 years, any sex	719 518		98	
	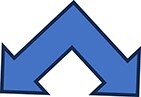		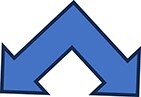	
	**CKD + CI**	**CKD no CI**	**CKD + CI**	**CKD no CI**
Dementia	9866	701 866	83	98
No dialysis	8582	566 839	81	98
No transplant	8184	523 772	80	98
Total	8184	523 772	80	98

CKD + CI: patients with CKD and mild CI/dementia.

CKD no CI: patients with CKD without mild CI/dementia.

After PSM, there were 8170 participants in both cohorts. The differences in comorbidities between the two groups were minor, and the populations were well matched, as shown by a standardized difference <0.1 (Table 2).

**Table 2:** Baseline demographics and comorbidities of the two cohorts before and after PSM.

	Before matching			After matching		
Variable	CKD with CI *n* = 8 173	CKD without CI *n* = 522 521	*P*-value	CKD with CI *n* = 8170	CKD without CI *n* = 8170	*P*-value
Age, years	60.5 ± 7.1	56.1 ± 9.6	<0.0001	60.5 ± 7.1	60.5 ± 6.6	0.8667
Gender male	4126 (50.5%)	275 675 (52.8%)	<0.0001	4124 (50.5%)	4186 (51.2%)	0.3242
Race and ethnicity						
White	4349 (53.2%)	281 220 (53.8%)	0.2739	4349 (53.2%)	464 (54.6%)	0.0711
Black or African American	2084 (25.5%)	116 597 (22.3%)	<0.0001	2083 (25.5%)	2125 (26.0%)	0.4524
Asian	196 (2.4%)	19 472 (3.7%)	<0.0001	196 (2.4%)	150 (1.8%)	0.0124
Hispanic or Latino Ethnicity	393 (4.8%)	33 665 (6.4%)	<0.0001	393 (4.8%)	379 (4.6%)	0.6057
Not Hispanic or Latino Ethnicity	5521(67.6%)	333 905 (63.9%)	<0.0001	5520 (67.6%)	5 670 (69.4%)	0.0115
Unknown ethnicity	2259 (27.6%)	154 951 (29.7%)	<0.0001	2257 (27.6%)	2 121 (26%)	0.0163
Comorbidities						
Endocrine	6473 (79.2%)	338 676 (64.8%)	<0.0001	6471 (79.2%)	6371 (78.0%)	0.0565
Circulatory system	6560 (70.3%)	342 547 (65.6%)	<0.0001	6558 (80.3%)	6413 (78.5%)	0.0050
Musculoskeletal	5311 (65.0%)	269 988 (51.7%)	<0.0001	5311 (65.0%)	5334 (65.3%)	0.7057
Digestive	5025 (61.5%)	232 630 (44.5%)	<0.0001	5025 (61.5%)	5047 (61.8%)	0.7234
Respiratory	4520 (55.3%)	205 655 (39.4%)	<0.0001	4519 (55.3%)	4679 (57.3%)	0.0116
Blood immune	3872 (47.4%)	147 560 (28.2%)	<0.0001	3870 (47.4%)	4008 (49.1%)	0.0307
Genitourinary	5579 (68.3%)	280 549 (53.7%)	<0.0001	5577 (68.3%)	5539 (67.8%)	0.5238
Nervous system	5964 (73.0%)	214 131 (41.0%)	<0.0001	5961 (73.0%)	5911 (72.4%)	0.3802
Neoplasms	2543 (31.1%)	129 114 (24.7%)	<0.0001	2543 (31.1%)	2587 (31.7%)	0.4583
Infectious diseases	3727 (45.6%)	142 977 (27.4%)	<0.0001	3725 (45.6%)	3880 (47.5%)	0.0151
Nicotine dependence	1976 (24.2%)	78 568 (15.0%)	<0.0001	1976 (24.2%)	2028 (24.8%)	0.3443
Injury (poisoning)	4327 (52.9%)	173 374 (33.2%)	<0.0001	4325 (52.9%)	4440 (54.3%)	0.0712
Mental behavioural	6254 (76.5%)	193 153 (37.0%)	<0.0001	6251 (76.5%)	6180 (75.6%)	0.1929
Factors influencing health status and contact with health services	6394 (78.2%)	337 254 (64%)	<0.001	6 393 (78.2%)	6288 (77.0%)	0.049
Medication						
Central nervous system	6461 (79.1%)	329 458 (63.1%)	<0.0001	6459 (79.1%)	6358 (77.8%)	0.0547
Cardiovascular system	6222 (76.1%)	339 775 (65.0%)	<0.0001	6221 (76.1%)	6118 (74.9%)	0.0609
Physical and laboratory parameters						
BMI	29.5 ± 7.3	31.7 ± 7.5	<0.0001	2618	2573	0.4497
Albumin, g/dL	3.7 ± 0.7	3.9 ± 0.6	<0.0001	3.7 ± 0.7	3.8 ± 0.6	0.790
Cholesterol, mg/dL	165.5 ± 52.1	176.0 ± 51.6	<0.0001	165 ± 52.1	171 ± 52.8	0.5514
Haemoglobin, g/dL	12.1 ± 2.2	12.6 ± 2.3	<0.0001	12.1 ± 2.2	12.4 ± 2.3	0.2169
eGFR, mL/min/1.73 m^2^	59.2 ± 21.44	50.9 ± 19.6	<0.0001	52.9 ± 21.4	51.4 ± 18.3	0.5389

Categorical values are expressed as numbers (percentages) and *P*-values by the *χ*^2^ test.

Continuous variables are expressed as mean (SD) and *P*-values by Student's *t*-test.

Data are presented as mean (SD) or *n* (%) as appropriate.

### Outcomes

**Figure 1:**
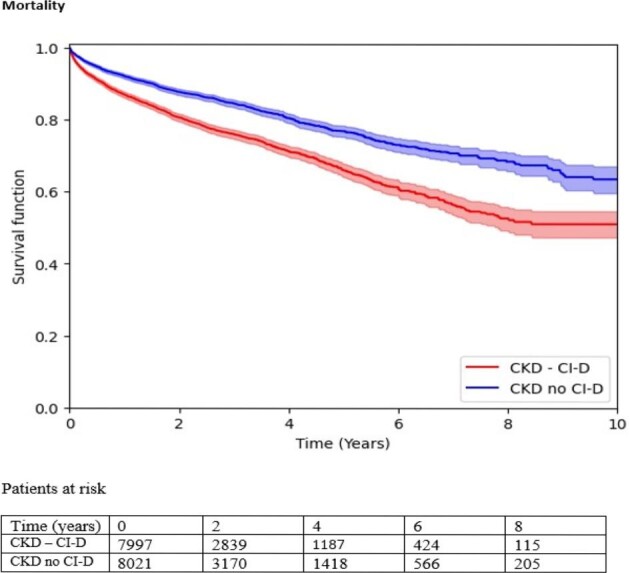
Survival function in the two groups over the space of 10 years.

We observed the outcomes over the following 10 years (Fig. 1). The median follow-up duration was 23.2 months for the CKD + CI cohort and 22.9 months for the CKD-no CI cohort. Statistical significance is indicated as **P < 0.001, and *P < 0.05.

CKD patients with concomitant CI had higher all-cause mortality (18.5% vs 12.6%), hazard ratio (HR) = 1.63 (1.50–1.76)**.

Patients affected by CI had a significant risk of developing an arrhythmia [14.5% vs 13.3%, HR = 1.26 (1.14–1.40)*], acute kidney injury (AKI) [19.5% vs 17.0%, HR = 1.25 (1.13–1.38)*] and hypotension [16.5% vs 12.5%, HR = 1.42 (1.27–1.59)**]. The two cohorts had the same risk of ischaemic heart disease [11.6 vs 11.5, HR = 1.10 (0.98–1.23)] and pulmonary embolism [1.9% vs 1.9%, HR = 1.08 (0.86–1.35)].

The risk of suffering transitory ischaemic attack was increased in patients with CKD + CI [2.7% vs 1.6%, HR = 1.86 (1.49–2.32)**; an increased risk of stroke was noted in this group: 11.3% vs 6.9%, HR = 1.89 (1.67–2.14)**]. In CKD + CI patients the risk of encephalopathy was higher [9.9% vs 5.0%, HR = 2.19 (1.93–2.50)**], as was the risk of epilepsy [4.3% vs 1.5%, HR = 3.26 (2.63–4.04)**].

More patients with CKD + CI were found to have malnutrition and sarcopenia [6.7% vs 4.00%, HR = 1.83 (1.58–2.12)**] and had a similar risk of developing type 2 diabetes [8.7% vs 10%, HR = 0.92 (0.80–1.07)] during follow-up. They had a greater likelihood of urinary tract infections (UTIs) [13.2% vs 9.3%, HR = 1.57 (1.41–1.75)**] and pneumonia [10.7% vs 7.9, HR = 1.54 (1.38–1.73)**].

Patients with CKD + CI had a higher likelihood of developing mood disorders [13.6% vs 9.7%, HR = 1.65 (1.45–1.88)**], anxiety disorders [13.0% vs 12.2%, HR = 1.23 (1.09–1.38)*], and psychotic episodes [9.8% vs 4.6%, HR = 2.45 (2.14–2.79)**] than patients with CKD without CI. However, the risk of alcohol dependence [1.7% vs 1.6%, HR = 1.14 (0.89–1.47)] and sleep disorders [11.7% vs 12.8%, HR = 1.06 (0.94–1.19)] was similar in the two cohorts.

The use of antidepressants [26.3% vs 16.3%, HR = 1.94 (1.76–2.14)**] and antipsychotic [18.6% vs 9.1%, HR = 2.38 (2.16–2.64)**], anticonvulsant [19.5% vs 15.1%, HR = 1.51 (1.37–1.66)**], and benzodiazepine medications [30.6% vs 26.6%, HR = 1.34 (1.22–1.46)**] was higher in patients with CKD + CI than in patients without CI. As expected, cholinesterase inhibitor drug use was almost exclusive to patients with CKD + CI [5.6% vs 0.1%, HR = 55.27 (28.55–107.03)**].

To confirm that the two groups of patients were homogeneous, we verified the use of renin–angiotensin–aldosterone inhibitors in the two populations, which was similar [19.3% vs 20.7%, HR 1.03 (0.93–1.13)].

Results are summarized in Table 3.

**Table 3:** Percentage of patients with outcome and hazard ratio (95% CI).

	CKD	CKD	Hazard ratio
Outcome	+ CI	no CI	(95% CI)
Mortality	18.5%	12.6%	1.63 (1.50–1.76)**
Cardio-renal events			
Ischaemic heart disease	11.6%	11.5%	1.10 (0.98–1.23)
Arrhythmia	14.5%	13.3%	1.26 (1.14–1.40)*
Pulmonary embolism	1.9%	1.9%	1.08 (0.86–1.35)
Acute kidney injury	19.5%	17.0%	1.25 (1.13–1.38)*
Hypotension	16.5%	12.5%	1.42 (1.27–1.59)**
Cerebrovascular events			
Stroke	11.3%	6.9%	1.89 (1.67–2.14)**
Transitory ischaemic attack	2.7%	1.6%	1.86 (1.49–2.32)**
Encephalopathy	9.9%	5.0%	2.19 (1.93–2.50)**
Epilepsy	4.3%	1.5%	3.26 (2.63–4.04)**
Metabolic events			
Malnutrition/sarcopenia	6.7%	4.0%	1.83 (1.58–2.12)**
Type 2 diabetes	8.7%	10.0%	0.92 (0.80–1.07)
Infective events			
Urinary tract infections	13.2%	9.3%	1.57 (1.41–1.75)**
Pneumonia	10.7%	7.9%	1.54 (1.38–1.73)**
Psychiatric events			
Mood disorders	13.6%	9.7%	1.65 (1.45–1.88)**
Anxiety	13.0%	12.2%	1.23 (1.09–1-38)*
Psychosis	9.8%	4.6%	2.45 (2.14–2.79)**
Sleep disorders	11.7%	12.8%	1.06 (0.95–1.19)
Alcohol abuse	1.7%	1.6%	1.14 (0.89–1.47)
Medication use			
Antidepressants	26.3%	16.3%	1.94 (1.76–2.14)**
Anticonvulsants	19.5%	15.1%	1.51 (1.37–1.66)**
Antipsychotics	18.6%	9.1%	2.38 (2.16–2.64)**
Anticholinesterase	5.6%	0.1%	55.28 (28.55–107.03)**
Benzodiazepines	30.6%	26.6%	1.34 (1.22–1.46)**
RAASis	19.3%	20.7%	1.03 (0.93–1.13)

**P < 0.001, *P < 0.05

RAASi, renin–angiotensin–aldosterone inhibitors.

## DISCUSSION

CKD has been associated with numerous comorbidity and complications, among which CI is particularly significant but often under-recognized [19]. This comorbidity is particularly concerning in older adults but is not exclusive to them. The implications of CI in CKD patients extend beyond the quality of life, influencing survival outcomes. Previous studies on CKD outcomes have traditionally focused on classic risk factors such as diabetes and hypertension, often overlooking the role of CI [20]. Moreover, most research has centred on older patients or those approaching dialysis, leaving a gap in understanding the cumulative risk associated with CI in younger patients with milder CKD. This study addresses this gap, demonstrating that even a short history of CI can increase the risk of death by nearly 50% in younger adult patients (60.5 ± 6.6 years old) with milder CKD (eGFR of 52.9 ± 21.4) in a 2-year time.

We demonstrated a higher risk of cerebrovascular disease, transitory ischaemic attack, hypotension, and AKI in patients with CKD + CI compared with patients with CKD alone.

The risk of stroke in patients with CKD (eGFR <60 mL/min) is 43% higher than in people with normal kidney function [21]. Having CI increases this risk by an additional 11.3%.

Several shared genetic pathways influence CKD and CI, particularly genes related to vascular health, inflammation, oxidative stress, and the renin–angiotensin system. These overlapping genetic factors suggest that people predisposed to one condition may also be at higher risk for the other, reinforcing the close connection between kidney and brain health [6].

CKD + CI have a higher risk of UTIs, an important finding in the context of increasing SGTL2 inhibitor use [22], a known risk factor for genitourinary infections. CKD + CI patients experience an increased risk of pneumonia, a leading cause of death in patients with dementia [23]. Dementia was linked to increased short-term mortality following pneumonia, particularly among antipsychotic users [24]. Patients with CI are more susceptible to infections, which often lead to hospitalization. Pneumonia and UTIs are the most common infections resulting in hospital visits. Additionally, infections can trigger delirium, which may worsen the symptoms of dementia [25]. Taking steps to prevent UTIs and pneumonia, especially in hospital settings, can significantly improve patient outcomes with CKD + CI [26].

Patients with CKD + CI exhibit a slightly higher risk of arrhythmias, which correlates with existing evidence highlighting an association between atrial fibrillation and cognitive decline [27].

The CKD + CI population had a greater prevalence of malnourishment or sarcopenia. The aetiological mechanisms are multifactorial; patients diagnosed with CKD may be subject to dietary restrictions, experience protein loss through proteinuria, and could be affected by diabetes, having specific dietary advice to avoid hyper/hypoglycaemia, all of which can lead to malnourishment and sarcopenia. Suffering from dementia can exacerbate nutritional risk due to increased resting energy expenditure and feeding difficulties [28–31].

We found that patients with CKD + CI have a higher risk of mood disorders, with a similar percentage to that reported in the literature (13.9%). Depressed CKD patients are often hospitalized and die sooner than those who are not depressed [32, 33]. Unsurprisingly, the use of antidepressant medication in CKD + CI was higher than in CKD alone. However, recent reports showed that these drugs have limited efficacy and constitute potential harm in CKD patients [34].

Patients diagnosed with CKD + CI carry a higher risk of psychotic symptoms, leading to worse cognitive function and representing both a prodrome and consequence of dementia [35, 36]. The situation can worsen with a decline in renal function, especially in the more advanced stages of CKD [37, 38]. Hence, these patients were more likely to receive antipsychotic medications [39], which increases the risk of AKI as kidney function declines [40].

We found a higher use of benzodiazepines in patients diagnosed with CKD + CI than in patients with CKD alone [41]. This is of concern, given that the use of benzodiazepines is, per se, associated with higher risk of dementia [42]. Only 5.6% of patients affected by CKD + CI were treated with cholinesterase inhibitors [43].

Encephalopathy, epilepsy, and the use of anticonvulsants were higher in the CKD + CI population than in CKD alone. The incidence of seizures is 5–10 times higher in patients with dementia [44]. Approximately 10% of patients with CKD can have seizures [45] and the use of antiepileptics can worsen cognition [39].

Damage to the brain appears to directly affect kidney function and promote the development of kidney disease, with bidirectional communication between the brain and kidneys [46, 47].

A cerebrovascular event in CKD creates additional risks due to contrast medium exposure, changes in blood pressure, reduced hydration, and nosocomial infections, which expose these patients to worsening of their renal function and death [48, 49].

This study presents the typical limitations of a retrospective study. It may be affected by all the biases occurring in data collection in the different databases and a lack of granularity in certain aspects of the data. However, its strengths are the vast real-world database from which CKD patient data were drawn and the PSM analysis, which controlled for many confounders and allowed comparison of CI patients with those CKD patients without this condition.

## CONCLUSIONS

Our study analysed many patients with real-world data originating from HCOs of multiple countries, in CKD patients with no history of dialysis or transplantation.

Our results showed how the combination of CKD and CI creates a greater risk of adverse events compared with patients who do not suffer from CI.

The study underscores the importance of a comprehensive cognitive assessment in CKD patients to identify the most vulnerable cohort at the earliest possible stage. Nephrologists must increase their confidence in managing therapies linked to cognitive decline in the context of CKD, and cooperation with psychiatry or neurology specialists should be encouraged to guarantee better care and quality of life in patients with CKD.

## Supplementary Material

sfae401_Supplemental_File

## Data Availability

The data supporting this study's findings are available from the TriNetX Analytics Network (https://trinetx.com).
