# Supplementary material for: Health outcomes in chronic kidney disease patients with cognitive impairment or dementia: a global collaborative analysis
Source: Clin Kidney J. 2024 Dec 11;18(1):sfae401. doi: 10.1093/ckj/sfae401 (PMC11761004; doi:10.1093/ckj/sfae401)
Supplement: sfae401_Supplemental_File [file sfae401_supplemental_file.docx]

## Cohorts’ definition

This section lists all terms used in the definitions of the two cohorts.

### Query Criteria for Cohort 1 (query name: CKD + CI)

This query was run on the Global Collaborative Network, and 115 HCOs were queried, and 115 HCOs responded. A total of 80 providers responded with patients. The final cohort included 8,184 patients who matched the query criteria listed in the table below.

|  | | | | | |
| --- | --- | --- | --- | --- | --- |
| Ungrouped terms | | | | | |
|  | must have |  | demographics | Age | Age (between 18 and 70 years (most recent occurrence)) |
|  |  | and any of | diagnosis | UMLS:ICD10CM:N18.3 | Chronic kidney disease, stage 3 (moderate) (between 18 and 70 years old at event) |
|  |  |  | diagnosis | UMLS:ICD10CM:N18.4 | Chronic kidney disease, stage 4 (severe) (between 18 and 70 years old at event) |
|  |  |  | diagnosis | UMLS:ICD10CM:N18.5 | Chronic kidney disease, stage 5 (between 18 and 70 years old at event) |
|  | cannot have |  | laboratory | TNX:8001 | Glomerular filtration rate/1.73 sq M.predicted [Volume Rate/Area] in Serum, Plasma or Blood by Creatinine-based formula (MDRD) (at most 5.00 mL/min/{1.73_m2} (most recent occurrence)) |
| Group 1 | | | | | |
|  | **Group 1A** | | | | |
|  | must have | any of | diagnosis | UMLS:ICD10CM:N18.3 | Chronic kidney disease, stage 3 (moderate) (between 18 and 70 years old at event) |
|  |  |  | diagnosis | UMLS:ICD10CM:N18.4 | Chronic kidney disease, stage 4 (severe) (between 18 and 70 years old at event) |
|  |  |  | diagnosis | UMLS:ICD10CM:N18.5 | Chronic kidney disease, stage 5 (between 18 and 70 years old at event) |
|  | date constraint | | The terms in this group occurred at any time | | |
|  | event relationship | | The first instance of Dementia occurred within 5 years on before the first instance of Group 1A | | |
|  | **Group 1B Dementia** | | | | |
|  | must have | any of | diagnosis | UMLS:ICD10CM:F03 | Unspecified dementia |
|  |  |  | diagnosis | UMLS:ICD10CM:F01 | Vascular dementia |
|  |  |  | diagnosis | UMLS:ICD10CM:F02 | Dementia in other diseases classified elsewhere |
|  |  |  | diagnosis | UMLS:ICD10CM:G31.84 | Mild cognitive impairment of uncertain or unknown etiology |
|  |  |  | diagnosis | UMLS:ICD10CM:G30 | Alzheimer's disease |
| Group 2 | | | | | |
|  | **Not on dialysis** | | | | |
|  | cannot have |  | procedure | UMLS:SNOMED:265764009 | Renal dialysis |
|  |  | or | diagnosis | UMLS:ICD10CM:T85.631 | Leakage of intraperitoneal dialysis catheter |
|  |  | or | diagnosis | UMLS:ICD10CM:T85.71 | Infection and inflammatory reaction due to peritoneal dialysis catheter |
|  |  | or | diagnosis | UMLS:ICD10CM:T85.691 | Other mechanical complication of intraperitoneal dialysis catheter |
|  |  | or | diagnosis | UMLS:ICD10CM:Z99.2 | Dependence on renal dialysis |
|  |  | or | procedure | UMLS:ICD10PCS:3E1M39Z | Irrigation of Peritoneal Cavity using Dialysate, Percutaneous Approach |
|  |  | or | diagnosis | UMLS:ICD10CM:T85.621 | Displacement of intraperitoneal dialysis catheter |
|  |  | or | diagnosis | UMLS:ICD10CM:Z49 | Encounter for care involving renal dialysis |
|  |  | or | diagnosis | UMLS:ICD10CM:T85.611 | Breakdown (mechanical) of intraperitoneal dialysis catheter |
|  |  | or | procedure | UMLS:ICD10PCS:5A1D | Physiological Systems / Performance / Urinary |
|  |  | or | procedure | UMLS:ICD9CM:39.95 | Hemodialysis |
|  |  | or | procedure | UMLS:CPT:1012740 | Dialysis Services and Procedures |
|  |  | or | diagnosis | UMLS:ICD10CM:Z91.15 | Patient's noncompliance with renal dialysis |
|  | date constraint | | The terms in this group occurred at any time | | |
| Group 3 | | | | | |
|  | **No Transplant** | | | | |
|  | cannot have |  | diagnosis | UMLS:ICD10CM:Z94.0 | Kidney transplant status |
|  |  | or | diagnosis | UMLS:ICD10CM:Z94.2 | Lung transplant status |
|  |  | or | diagnosis | UMLS:ICD10CM:Z94.1 | Heart transplant status |
|  |  | or | diagnosis | UMLS:ICD10CM:Z94.3 | Heart and lungs transplant status |
|  |  | or | diagnosis | UMLS:ICD10CM:Z94.4 | Liver transplant status |
|  |  | or | diagnosis | UMLS:ICD10CM:Z94.83 | Pancreas transplant status |
|  |  | or | procedure | UMLS:ICD10PCS:0BY | Transplantation |
|  |  | or | procedure | UMLS:ICD10PCS:0TY | Transplantation |
|  |  | or | procedure | UMLS:ICD10PCS:02Y | Transplantation |
|  |  | or | procedure | UMLS:ICD10PCS:0FY | Transplantation |
|  |  | or | procedure | UMLS:CPT:1006036 | Lung Transplantation Procedures |
|  |  | or | procedure | UMLS:CPT:1008098 | Renal Transplantation Procedures |
|  |  | or | procedure | UMLS:CPT:1006332 | Heart/Lung Transplantation Procedures |
|  |  | or | procedure | UMLS:CPT:1007811 | Liver Transplantation Procedures |
|  |  | or | procedure | UMLS:CPT:1007944 | Pancreas Transplantation Procedures |
|  |  | or | procedure | UMLS:SNOMED:88039007 | Transplant of lung |
|  |  | or | procedure | UMLS:SNOMED:70536003 | Transplant of kidney |
|  |  | or | procedure | UMLS:SNOMED:32413006 | Transplantation of heart |
|  |  | or | procedure | UMLS:SNOMED:232973007 | Allotransplant of heart |
|  |  | or | procedure | UMLS:SNOMED:174802006 | Allotransplant of heart and lung |
|  |  | or | procedure | UMLS:SNOMED:18027006 | Transplantation of liver |
|  |  | or | procedure | UMLS:SNOMED:62438007 | Transplantation of pancreas |
|  | date constraint | | The terms in this group occurred at any time | | |

### Query Criteria for Cohort 2 (query name: CKD no CI)

This query was run on the Global Collaborative Network, and 115 HCOs were queried, and 115 HCOs responded. A total of 98 provider(s) responded with patients. The final cohort included 523,772 patients who matched the query criteria listed in the table below.

| Ungrouped terms | | | | | |
| --- | --- | --- | --- | --- | --- |
|  | must have |  | demographics | Age | Age (between 18 and 70 years (most recent occurrence)) |
|  |  | and any of | diagnosis | UMLS:ICD10CM:N18.3 | Chronic kidney disease, stage 3 (moderate) (between 18 and 70 years old at event) |
|  |  |  | diagnosis | UMLS:ICD10CM:N18.4 | Chronic kidney disease, stage 4 (severe) (between 18 and 70 years old at event) |
|  |  |  | diagnosis | UMLS:ICD10CM:N18.5 | Chronic kidney disease, stage 5 (between 18 and 70 years old at event) |
|  | cannot have |  | laboratory | TNX:8001 | Glomerular filtration rate/1.73 sq M.predicted [Volume Rate/Area] in Serum, Plasma or Blood by Creatinine-based formula (MDRD) (at most 5.00 mL/min/{1.73_m2} (most recent occurrence)) |
| Group 1 | | | | | |
|  | **Dementia** | | | | |
|  | cannot have |  | diagnosis | UMLS:ICD10CM:G31.84 | Mild cognitive impairment of uncertain or unknown etiology |
|  |  | or | diagnosis | UMLS:ICD10CM:G30 | Alzheimer's disease |
|  |  | or | diagnosis | UMLS:ICD10CM:F02 | Dementia in other diseases classified elsewhere |
|  |  | or | diagnosis | UMLS:ICD10CM:F03 | Unspecified dementia |
|  |  | or | diagnosis | UMLS:ICD10CM:F10.27 | Alcohol dependence with alcohol-induced persisting dementia |
|  |  | or | diagnosis | UMLS:ICD10CM:G31.0 | Frontotemporal dementia |
|  |  | or | diagnosis | UMLS:ICD10CM:F01 | Vascular dementia |
|  | date constraint | | The terms in this group occurred at any time | | |
| Group 2 | | | | | |
|  | **Not on dialysis** | | | | |
|  | cannot have |  | procedure | UMLS:SNOMED:265764009 | Renal dialysis |
|  |  | or | diagnosis | UMLS:ICD10CM:T85.631 | Leakage of intraperitoneal dialysis catheter |
|  |  | or | diagnosis | UMLS:ICD10CM:T85.71 | Infection and inflammatory reaction due to peritoneal dialysis catheter |
|  |  | or | diagnosis | UMLS:ICD10CM:T85.691 | Other mechanical complication of intraperitoneal dialysis catheter |
|  |  | or | diagnosis | UMLS:ICD10CM:Z99.2 | Dependence on renal dialysis |
|  |  | or | procedure | UMLS:ICD10PCS:3E1M39Z | Irrigation of Peritoneal Cavity using Dialysate, Percutaneous Approach |
|  |  | or | diagnosis | UMLS:ICD10CM:T85.621 | Displacement of intraperitoneal dialysis catheter |
|  |  | or | diagnosis | UMLS:ICD10CM:Z49 | Encounter for care involving renal dialysis |
|  |  | or | diagnosis | UMLS:ICD10CM:T85.611 | Breakdown (mechanical) of intraperitoneal dialysis catheter |
|  |  | or | procedure | UMLS:ICD10PCS:5A1D | Physiological Systems / Performance / Urinary |
|  |  | or | procedure | UMLS:ICD9CM:39.95 | Hemodialysis |
|  |  | or | procedure | UMLS:CPT:1012740 | Dialysis Services and Procedures |
|  |  | or | diagnosis | UMLS:ICD10CM:Z91.15 | Patient's noncompliance with renal dialysis |
|  | date constraint | | The terms in this group occurred at any time | | |
| Group 3 | | | | | |
|  | **No Transplant** | | | | |
|  | cannot have |  | diagnosis | UMLS:ICD10CM:Z94.0 | Kidney transplant status |
|  |  | or | diagnosis | UMLS:ICD10CM:Z94.2 | Lung transplant status |
|  |  | or | diagnosis | UMLS:ICD10CM:Z94.1 | Heart transplant status |
|  |  | or | diagnosis | UMLS:ICD10CM:Z94.3 | Heart and lungs transplant status |
|  |  | or | diagnosis | UMLS:ICD10CM:Z94.4 | Liver transplant status |
|  |  | or | diagnosis | UMLS:ICD10CM:Z94.83 | Pancreas transplant status |
|  |  | or | procedure | UMLS:ICD10PCS:0BY | Transplantation |
|  |  | or | procedure | UMLS:ICD10PCS:0TY | Transplantation |
|  |  | or | procedure | UMLS:ICD10PCS:02Y | Transplantation |
|  |  | or | procedure | UMLS:ICD10PCS:0FY | Transplantation |
|  |  | or | procedure | UMLS:CPT:1006036 | Lung Transplantation Procedures |
|  |  | or | procedure | UMLS:CPT:1008098 | Renal Transplantation Procedures |
|  |  | or | procedure | UMLS:CPT:1006332 | Heart/Lung Transplantation Procedures |
|  |  | or | procedure | UMLS:CPT:1007811 | Liver Transplantation Procedures |
|  |  | or | procedure | UMLS:CPT:1007944 | Pancreas Transplantation Procedures |
|  |  | or | procedure | UMLS:SNOMED:88039007 | Transplant of lung |
|  |  | or | procedure | UMLS:SNOMED:70536003 | Transplant of kidney |
|  |  | or | procedure | UMLS:SNOMED:32413006 | Transplantation of heart |
|  |  | or | procedure | UMLS:SNOMED:232973007 | Allotransplant of heart |
|  |  | or | procedure | UMLS:SNOMED:174802006 | Allotransplant of heart and lung |
|  |  | or | procedure | UMLS:SNOMED:18027006 | Transplantation of liver |
|  |  | or | procedure | UMLS:SNOMED:62438007 | Transplantation of pancreas |
|  | date constraint | | The terms in this group occurred at any time | | |
